# Subclinical Cardiac Dysfunction in Childhood Cancer Survivors on 10-Years Follow-Up Correlates With Cumulative Anthracycline Dose and Is Best Detected by Cardiopulmonary Exercise Testing, Circulating Serum Biomarker, Speckle Tracking Echocardiography, and Tissue Doppler Imaging

**DOI:** 10.3389/fped.2020.00123

**Published:** 2020-03-31

**Authors:** Cordula Maria Wolf, Barbara Reiner, Andreas Kühn, Alfred Hager, Jan Müller, Christian Meierhofer, Renate Oberhoffer, Peter Ewert, Irene Schmid, Jochen Weil

**Affiliations:** ^1^Department of Congenital Heart Disease and Pediatric Cardiology, German Heart Center Munich, Technical University Munich, Munich, Germany; ^2^Faculty of Sport and Health Sciences, Institute of Preventive Pediatrics, Technical University Munich, Munich, Germany; ^3^Department of Pediatric Hematology and Oncology, Dr. von Hauner Children's Hospital, Ludwig-Maximilians-University Munich, Munich, Germany

**Keywords:** cardiotoxicity, childhood cancer, biomarker, speckle tracking, tissue doppler, anthracycline, cardiovascular, subclinical

## Abstract

**Background:** Survivors of childhood cancer are at risk for anthracycline- and/or radiotherapy-induced cardiotoxicity.

**Aims:** The aim of this study was to assess clinical, laboratory, and imaging parameters of subclinical cardiovascular disease in childhood cancer survivors.

**Methods:** Patients underwent cardiopulmonary exercise test (CPET), laboratory testing, transthoracic echocardiography (TTE) with tissue doppler imaging (TDI) and speckle tracking. A subset of patients also underwent cardiovascular magnetic resonance imaging (CMR). Findings were correlated to cumulative anthracycline and exposure to mediastinal irradiation during cancer treatment. In a subgroup analysis, TTE and CMR findings were compared to data from 40 gender- and age-matched patients with childhood onset hypertrophic cardiomyopathy (HCM).

**Results:** Cardiac evaluation was performed in 79 patients (43 males) at 11.2 ± 4.5 years after cancer treatment. Oncologic diagnosis at a median age of 12.0 years was Hodgkin lymphoma in 20, sarcoma in 17, acute leukemia in 24, relapse leukemia in 10, and others in 8 patients. Cumulative anthracycline dose exceeded 300 mg/m^2^ in 28 patients. Twenty six patients also received mediastinal irradiation. Decreased peak respiratory oxygen uptake in % predicted on CPET, increased levels of N-terminal pro-brain natriuretic peptide (NTproBNP), increased global longitudinal strain on TTE speckle tracking, and diastolic dysfunction on TDI were the most prominent findings on detailed cardiology follow-up. In contrast to HCM patients, childhood cancer survivors did not show left ventricular hypertrophy (LVPWd z-score median 0.9 vs. 2.8, *p* < 0.001), hyperdynamic systolic function on TTE (Ejection fraction 62 ± 7 vs. 72 ± 12%, *p* = 0.001), or fibrotic myocardial changes on CMR (Late gadolinium positive 0/13 vs. 13/36, *p* = 0.001; extracellular volume fraction 22 ± 2 vs. 28 ± 3, *p* < 0.001) at time of follow-up. There was no correlation between chest radiation exposure and abnormal cardiac findings. Cumulative anthracycline dose was the only significant independent predictor on multivariate analysis for any cardiovascular abnormality on follow-up (*p* = 0.036).

**Conclusion:** Increasing cumulative anthracycline dose during cancer treatment correlates with subclinical cardiac dysfunction in childhood cancer survivors best detected by elevated cardiac serum biomarkers, decreased exercise capacity on CPET, and abnormalities on echocardiographic speckle tracking and TDI.

## Introduction

Due to advancements in treatment, survival rates for childhood cancer have substantially improved during the past decades with an overall 5-year survival rate of over 80% today (1–3). More than half of childhood cancer patients receive anthracyclines as part of their cancer therapy (4) and anthracycline-mediated cardiovascular complications contribute to morbidity and mortality in the growing population of childhood cancer survivors [reviewed in Franco et al., (5), Raj et al., (6), Todaro et al., (7), Van Der Pal et al., (8) and (9)]. Anthracyclines express their antiproliferation of cancer cell effects through inhibition of DNA replication, RNA replication, DNA cross-linking, and topoisomerases (10). The pathophysiology of anthracycline mediated cardiotoxicity is multifactorial and involves multiple mechanisms. Oxidative stress (11–14), induction of apoptosis, formation of toxic metabolites, altered cardiomyocyte gene expression, transcription and translation (15–18), as well as impaired oxidative phosphorylation and adenosine triphosphate (ATP) synthesis (19), have been suggested to play a major role in anthracycline-induced cardiotoxicity. Survivors of childhood cancer are especially vulnerable to these risks given their young age at the time of treatment and the exposure of cardiotoxicity of their growing and developing heart. Exposure to mediastinal irradiation might contribute additionally to cardiotoxic effects (8, 20–24). Cardiotoxicity can manifest at an early stage of therapy (during or immediately after treatment) (25) or many years thereafter (9, 26). The clinical presentation of childhood cancer survivors suffering from cancer-treatment related cardiotoxicity varies from asymptomatic cardiac dysfunction to overt congestive heart failure (8, 9, 20). Other cardiovascular sequelae include coronary artery disease, stroke, arrhythmias, and valvular and vascular dysfunction (20). There is now a growing body of evidence that genetic risk factors might contribute to the large variability of cardiotoxic disease in individuals (27–30).

The primary prevention of long-term adverse effects of childhood cancer treatment includes modification of therapeutic exposures (31–33), the concomitant use of cardioprotective therapies (34–37), or the use of alternative chemotherapeutics with maximal efficacy and minimal short- and long-term adverse effects (36).

Secondary prevention after completion of anticancer treatment, such as improved surveillance, appropriate screening strategies, and preventative therapies for cardiovascular complications, require an interdisciplinary approach between oncologists and cardiologist.

Standardized clinical evaluation with regular follow-up and assessment of the entire cardiovascular system is necessary in childhood cancer survivors (38). However, even though consensus-based guidelines for cardiotoxicity monitoring following chemotherapy using echocardiography exist (38), the optimal timing and cost-effectiveness for such monitoring needs further investigation (39, 40). Identification of subclinical cardiotoxicity can be challenging (41).

Data are scarce with regards to the optimal use of serum biomarkers, the mode and sensitivity of distinct imaging modalities, and optimal management strategies once subclinical cardiotoxicity has been detected, specifically in children.

The aim of the study was to assess laboratory, cardiopulmonary exercise testing, echocardiographic, and cardiac magnetic resonance imaging findings in clinically asymptomatic childhood cancer survivors on long-term follow-up. The identification of monitoring parameters shall optimize risk stratification, identify subclinical cardiovascular disease, and enable preventative treatment measures in time before overt clinical disease in childhood cancer survivors becomes evident.

## Materials and Methods

### Study Design

In this retrospective single tertiary care center study, medical charts were reviewed from all childhood cancer survivors followed at the pediatric cardiology outpatient unit at the German Heart Center Munich between March 2015 and October 2018. Demographic, clinical, laboratory, transthoracic echocardiographic (TTE), cardiopulmonary exercise testing (CPET), cardiovascular magnetic resonance (CMR), and other patient-related data were collected retrospectively by review of patients' charts at last follow-up.

### Patient Population

Data from a total of 79 patients with a primary diagnosis of childhood cancer and initial cancer treatment between 0 and 18 years of age were analyzed. For subgroup analysis, TTE and CMR findings were compared with 40 gender- and age-matched patients with childhood onset hypertrophic cardiomyopathy (HCM). The diagnosis of HCM was based on clinical evaluation and cardiac catheterization or TTE detecting myocardial hypertrophy (defined as a z-score of >2) in the absence of another cardiac or systemic disease causing the degree of left ventricular hypertrophy identified (42, 43). This patient cohort was selected for comparison to evaluate similarities and differences between cardiomyopathic changes according to underlying etiology. Data were collected as previously described (44).

### Cardiopulmonary Exercise Testing

CPET was performed in sitting position on a bike-ergometer. A standardized testing protocol was performed and physical working capacity, peak oxygen uptake (VO_2_ max), evidence of myocardial ischemia or arrhythmia and escalation of heart rate or blood pressure during or after exertion were recorded (45). For the peak respiratory oxygen uptake in % predicted [VO_2_ max (% norm)], a cut-off level of <80 was determined as “abnormal.”

### Serum Biomarkers

The serum biomarkers cardiac troponin T (cTNT) and N-terminal pro-brain natriuretic peptide (NTproBNP) levels were determined as marker for early systolic dysfunction or myocardial stress. Values >0.01 ng/dL and >100 ng/dL, respectively, were considered as “elevated.”

### Transthoracic Echocardiography

Echocardiograms were obtained at the time of outpatient visits and were remeasured by blinded study staff (AK). TTE was performed using standard equipment in routine clinical practice and using standard views according to the American Society of Echocardiography guidelines (46–48). Echopac Software (General Electric, Vingmed, Horten, Norway) was used for offline analysis. Measurements of left ventricular wall thickness and enddiastolic ventricular diameters were standardized to body surface area and given as z-scores [http://parameterz.blogspot.com/2008/09/z-scores-of-cardiac-structures.html, (49)]. Z-scores of <-2 or > +2 are considered as “abnormal,” myocardial hypertrophy is defined as a z-score of >2 (42). Global longitudinal strain (GLS) was assessed by speckle tracking and diastolic function by tissue and pulse wave Doppler echocardiography on apical four-chamber views at the lateral and septal mitral valve annulus as previously described (48, 50). Reference values for normal and abnormal measurements were extracted from literature of large gender- and age-matched cohorts with similar ethnicity (49, 51–53). An ejection fraction (EF) of <50 (51) and GLS >-20 (53, 54) were considered as abnormal. Left atrial (LA) volume standardized to body surface area (BSA) of >34 ml/m^2^ was considered as enlarged (51). Mitral valve E/A ratio of >2 and a septal E/E' ratio of >8 was considered as abnormal (52, 55).

### Cardiovascular Magnetic Resonance Tomography

CMR was performed on a 1.5 Tesla MR scanner (Magnetom Avanto, Siemens Healthcare, Software Version VD13, Erlangen, Germany). Cine images (balanced steady-state free precession) were acquired in short axis and four chamber orientations in breath hold to evaluate the ventricular volume, ventricular mass, ejection fraction, and regional wall anomalies. Late Gadolinium Enhancement (LGE) was acquired using a T1-weighted phase-sensitive inversion recovery sequence 10 min after intravenous administration of an extracellular MR contrast agent (Gadopentetat) to detect focal fibrosis (56).

Native and post contrast T1 mapping, using a modified look-locker inversion recovery sequence (MOLLI) (57), with non-rigid motion correction reconstruction, were assessed in short axis and four chamber orientation. Image quality was assessed by revising T1 maps and error maps of the region of interest. Extracellular volume (ECV) was calculated as previously described (58). An ECV >25 was considered as abnormal.

### Ethics

The study was conducted in accordance with the Declaration of Helsinki (revision 2008) and the Good Clinical Practice guidelines. The study protocol was approved by the local ethical board of the Technical University Munich (ethical approval number 243/17S, 10/16/2017).

### Statistics

Data are presented as mean and standard deviation or as median and minimum / maximum according to distribution. Kolmogorov-Smirnoff Test was used to assess distribution of variances. Independent *t*-Test and Mann-Whitney-Test were used according to data distribution to compare parameters between the two groups. ANOVA (analysis of variances) or Kruskall-Wallis-test were used to compare differences between multiple groups (oncologic diagnosis) according to data distribution. Fisher's Test or Pearson chi-square was used for comparison of categorical variables. Pearson correlation was used to assess the correlation between anthracycline dose, radiation exposure, age at cancer treatment, age at follow-up, follow-up time, and cardiovascular findings. Binary outcome variables (abnormal or normal) were created using cut-off for the respective variables. Backward multivariate binary logistic regression analysis was done for the categorical variable “Any cardiovascular abnormality” defined as decreased exercise capacity, NTproBNP level, or increased septal E/E' ratio on TDI as dependent variable and cumulative anthracycline dose, radiation exposure, gender, age at cancer treatment, age at follow-up, and follow-up time as covariates. Cut-off levels of cumulative anthracycline dose predictive for any cardiovascular abnormality on follow-up were derived from the Receiver operating characteristic (ROC) curve. The significance level of *p*-value was set at <0.05. Data were analyzed on IBM SPSS Version 25.

## Results

### Patient Characteristics

Demographic and clinical patient characteristics are summarized in Table 1. A detailed cardiac evaluation was performed in 79 patients (43 males) at 11.2 ± 4.5 years after cancer treatment. Underlying oncologic diagnosis was acute (*N* = 24) or relapse leukemia (*N* = 10), Hodgkin lymphoma (*N* = 20), sarcoma (*N* = 17), nephroblastoma (*N* = 4), neuroblastoma (*N* = 3), and hepatoblastoma (*N* = 1). Median age at primary oncologic diagnosis was 12.0 years. Mean cumulative anthracycline dose was 261±104 mg/m^2^ and exceeded 300 mg/m^2^ in 36% of patients. Patients with sarcomas received significantly higher doses of anthracycline compared to the remaining patients. Twenty-six patients, most of them with Hodgkin lymphomas and relapse ALL, also received mediastinal irradiation with heart exposure. All patients with relapse ALL and one patient with ALL received mediastinal irradiation in the setting of total body irradiation for preparation to bone marrow transplant (Table 1). Cumulative ionizing radiation dose was <30 Gray in all these patients.

**Table 1:** Patient characteristics.

**Patient characteristics**	**Total** ***N* = 79**	**Hodgkin lymphoma** ***N* = 20**	**Sarcoma** ***N* = 17**	**ALL** ***N* = 24**	**Relapse ALL** ***N* = 10**	Others^*^ N = 8	***p***
Male *n/N* (%)	43/79 (54)	11/20 (55)	10/17 (59)	14/24 (58)	3/10 (30)	5/8 (22)	ns^a^
Age at diagnosis (years)	12.0 [0.2–17.9]	14.0 [6.7–17.9]	12.5 [3.8–17.7]	8.5 [2.7–16.7]	6.3 [2.0–11.4]	5.0 [0.2–13.7]	<0.001^b^
Age at follow-up (years)	20.9 [11.9–32.0]	23.2 [18.8–30.2]	19.9 [17.0–27.6]	20.3 [11.9–24.8]	19.5 [17.1–26.1]	21.0 [13.9–32.0]	<0.001^b^
Follow-up time (years)	11.2 ± 4.5	10.1 ± 3.1	8.2 ± 3.1	12.0 ± 4.5	13.4 ± 2.8	15.7 ± 6.8	<0.001^b^
Height (cm)	169 ± 10	172 ± 8	171 ± 10	170 ± 9	159 ± 10	167 ± 14	0.013^b^
Weight (kg)	68 ± 17	78 ± 23	64 ± 9	70 ± 11	53 ± 13	62 ± 19	0.002^b^
BMI	22.9 [14.1–41.5]	25.0 [18.2–41.5]	21.3 [18.6–29.4]	23.2 [17.6–35.5]	20.1 [17.3–27.5]	22.4 [14.1–26.9]	0.017^b^
BSA (m^2^)	1.77 ± 0.26	1.91 ± 0.31	1.74 ± 0.15	1.8 ± 0.16	1.53 ± 0.23	1.69 ± 0.33	0.001^b^
Anthracycline dose (mg/m^2^)	261 ± 104	187 ± 75	367 ± 87	225 ± 68	290 ± 8	283 ± 119	<0.001^b^
<100 mg/m^2^, *n/N* (%)	3/79 (4)	1/20 (5)	1/17 (6)	1/24 (4)	0/10	0	<0.001^a^
100–199 mg/ m^2^, *n/N* (%)	24/79 (31)	15/20 (79)	0	6/24 (25)	2/10 (20)	2/8 (25)	
200–299 mg/m^2^, *n/N* (%)	23/79 (29)	1/20 (5)	0	16/24 (68)	3/10 (30)	3/8 (38)	
>300 mg/m^2^, *n/N* (%)	28/79 (36)	3/20 (16)	16/17 (94)	1/24 (4)	5/10 (50)	3/8 (38)	
Mediastinal irradiation	26/79 (33)	14/20 (70)	0	1/24 (4)	10/10 (100)	1/8 (13)	<0.001^a^
Total body irradiation	11/79 (14)	0	0	1/24 (4)	10/10 (100)	0	<0.001^a^

*Data are shown as mean ± standard deviation or median [range] according to data distribution*.

* *Other oncologic diagnosis include nephroblastoma (N = 4), neuroblastoma (N = 3), and hepatoblastoma (N = 1)*.

a *Pearson chi square*.

b *ANOVA (analysis of variances) or Kruskall-Wallis-test according to data distribution*.

N, number of patients; ns, not significant; mg, milligram; cm, centimeter.

### Cardiovascular Findings in Childhood Cancer Survivors

**Figure 1:**
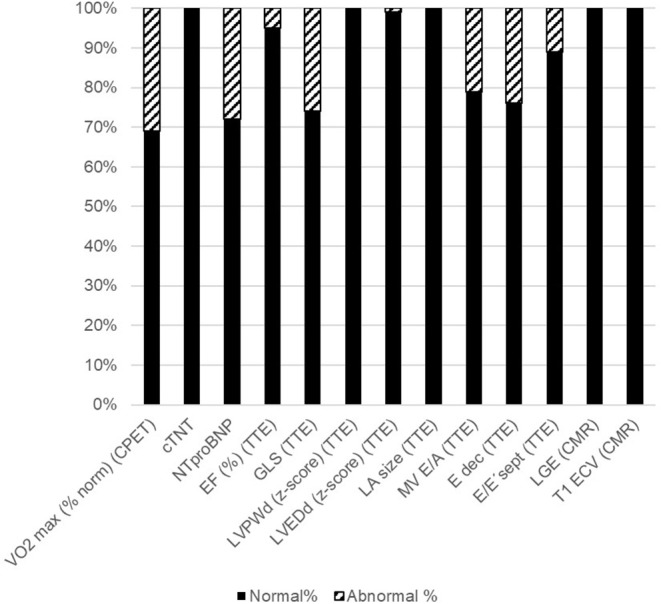
Abnormal cardiovascular parameters. Shown are the percentages of childhood cancer survivors with normal cardiovascular parameters (black bars) and those with abnormal cardiovascular parameters (striped bars). Please see methods section for cut-off values. CPET: cardiopulmonary exercise testing; VO_2_ max (% norm): peak respiratory oxygen uptake in % predicted; EF, Ejection Fraction; GLS, Global longitudinal strain; LVPWd, Enddiastolic left ventricular posterior wall dimension; LVEDd, Left ventricular enddiastolic diameter; LA, left atrium; MV, Mitral valve; CMR, cardiovascular magnetic resonance imaging; LGE, Late gadolinium enhancement; ECV, extracellular volume fraction.

All patients were clinically asymptomatic and in NYHA functional class I at the time of follow-up. Abnormal findings on detailed cardiovascular examination in childhood cancer survivors included decreased exercise capacity on CPET, increased NTproBNP levels on laboratory testing, impaired global longitudinal strain on TTE speckle tracking, and evidence of diastolic dysfunction on tissue Doppler echocardiography (Figure 1). No patient had a medical history or clinical evidence of significant pulmonary disease. Decreased exercise capacity as shown by a peak respiratory oxygen uptake in % predicted (VO_2_ max) of <80 on CPET was evident in a third of patients. Cardiac troponin T levels were normal in all patients. NTproBNP levels >100 ng/dL were measured in 29% of childhood cancer survivors. Whereas, left ventricular systolic function (EF) and left ventricular dimensions were normal on TTE in most patients, there were abnormal GLS measurements in off-line echocardiographic speckle tracking analysis in about 28% of patients. No left atrial enlargement was documented on TTE, but pulse wave and tissue Doppler examination showed signs of diastolic dysfunction in 21, 24, and 11%, respectively (abnormal MV E/A ratio, E deceleration time and septal E/E'. There was no evidence of focal (LGE positive) or interstitial (increased ECV on T1 map) myocardial fibrosis on CMR imaging (Figure 1).

TTE and CMR data of childhood cancer survivors were compared to age- and gender-matched patients with an underlying diagnosis of childhood hypertrophic cardiomyopathy (HCM) secondary to mutations in sarcomere genes. The majority of HCM patients carried a pathogenic variant in either the *MYH7* (14/40, 35%) or the *MYBPC3* (12/40, 30%) gene. Other genes affected included *TNNT2, TNNI3, ML2, TPM1* in 7 of 40 HCM patients (17.5%) and multiple mutations were detected in 4 of 40 HCM patients (10 %). Molecular diagnostic testing was negative in 3 of 40 HCM patients (7.5 %). Left ventricular outflow tract obstruction was present in 14 of 40 HCM patients (35 %). In contrast to HCM patients, childhood cancer survivors did not show left ventricular hypertrophy or hyperdynamic systolic function on TTE (Table 2). Fibrotic myocardial changes, such as focal fibrosis assessed by the presence of LGE on CMR and interstitial fibrosis assessed by increased ECV on CMR T1 map, were observed in a subset of HCM patients but not in childhood cancer survivors at the time of follow-up (Table 2). Evidence of diastolic dysfunction on echocardiographic pulse wave and tissue Doppler was observed in both groups, but only HCM and not childhood cancer survivor patients presented with enlarged standardized left atrial volumes (Table 2). Enlarged left atria documented in HCM patients occurred independently from moderate mitral regurgitation noted in only 3 of the 10 HCM patients with enlarged left atria. No higher degree valve stenosis was noted in HCM patients.

**Table 2:** Cardiovascular findings of childhood cancer survivors compared to childhood hypertrophic cardiomyopathy patients.

**Clinical findings**	**Total** ***N* = 120**	**Childhood cancer survivors** ***N* = 79**	**Childhood hypertrophic cardiomyopathy** ***N* = 40**	***p***
Male; *n/N* (%)	67/120 (56)	43/79 (54)	24/40 (60)	ns^a^
Age at diagnosis (years)	10.7 [0–18.9]	12.0 [0.2–17.9]	6.0 [0.0–18.9]	0.024^b^
Age at follow-up (years)	20.1 [0.4–51.2]	20.9 [11.9–32.0]	17.7 [0.4–51.2]	0.044^b^
Follow-up time (years)	11.1 ± 6.2	11.2 ± 4.5	10.8 ± 8.6	ns
**CPET**				
VO_2_ max (% norm)	87 ± 21	91 ± 21	78 ± 17	0.003^b^
**TTE**				
LVPWd (z-score)	1.0 [−0.8 to 7.1]	0.9 [−0.8 to 1.9]	2.8 [−0.4 to 7.1]	<0.001^b^
EF (%)	64 ± 9	62 ± 7	72 ± 12	0.001^b^
GLS	−18.0 [−27.2 to 5.0]	−18.7 [−22.6 to 10.0]	−13.1 [−27.2 to 4.8]	0.007^b^
Enlarged LA; *n/N (%)*	10/109 (9)	0	10/38 (26)	<0.001^a^
MV E/A	1.6 ± 0.5	1.6 ± 0.5	1.6 ± 0.7	ns^b^
E dec	175 ± 41	175 ± 37	172 ± 52	ns^b^
E/E' sept	6.9 ± 2.8	6.4 ± 2.3	9.1 ± 3.5	0.009^b^
**CMR**				
LGE positive; *n/N* (%)	13/36 (36)	0	13/36 (36)	0.001^a^
T1 ECV	25 ± 4	22 ± 2	28 ± 3	<0.001^b^

*Data are shown as mean ± standard deviation or median [range] according to data distribution*.

a *Fishers exact Test*.

b *independent t-Test or Mann-Whitney-Test according to data distribution*.

*n/N*, number of patients; ns, not significant; CPET, cardiopulmonary exercise testing; VO_2_ max (% norm), peak respiratory oxygen uptake in % predicted; TTE, transthoracic echocardiography; EF, Ejection Fraction; GLS, Global longitudinal strain; LVPWd, Enddiastolic left ventricular posterior wall dimension; LA, left atrium; MV, Mitral valve; CMR, cardiovascular magnetic resonance imaging; LGE, Late gadolinium enhancement; ECV, extracellular volume fraction.

### Univariate Analysis of Risk Factors for Abnormal Clinical Features

Patients were grouped into five categories according to oncologic diagnosis and variance analysis was carried out to assess if there were differences of abnormalities in cardiovascular findings between groups. There were significant differences between oncologic groups concerning decreased exercise capacity, NTproBNP levels and evidence of diastolic dysfunction on echocardiographic tissue Doppler imaging (mitral valve septal E/E'). Elevated NTproBNP levels and evidence for diastolic dysfunction were mostly observed in patients treated for sarcoma (Table 3).

**Table 3:** Cardiovascular findings according to oncologic diagnosis.

**Cardiovascular parameters**	**Total** ***N* = 79**	**Hodgkin lymphoma** ***N* = 20**	**Sarcoma** ***N* = 17**	**ALL** ***N* = 24**	**Relapse ALL** ***N* = 10**	Others^*^ N = 8	***p***
**CPET**							
VO_2_ max (% norm)	91 ± 21	94 ± 26	79 ± 13	96 ± 19	89 ± 20	92 ± 24	ns^d^
**Laboratory**							
NTproBNP (ng/dL)	40 [6-763]	40 [6-168]	130 [17-763]	33 [8-77]	37 [7-139]	54 [15-273]	0.043^d^
NTproBNP elevated^a^	18/65 (28)	5/19 (26)	10/15 (67)	0/17	2/9 (22)	1/5 (20)	0.001^e^
*n/N* (%)							
cTNT (ng/dL)	0.004 [0.001-0.10]	0.004 [0.003-0.10]	0.006 [0.001-0.009]	0.003 [0.003-0.008]	0.005 [0.003-0.01]	0.003 [0.003-0.006]	ns^*d*^
**TTE morphology**							
Myocardial hypertrophy^b^;	0/79	0/20	0/17	0/24	0/10	0/8	NA
*n/N* (%)							
LV dilatation^c^	1/79 (1)	0/20	1/17 (6)	0/24	0/10	0/8	ns^e^
*n/N* (%)							
**TTE systolic function**							
EF (%)	62 ± 7	63 ± 7	60 ± 8	63 ± 7	65 ± 8	61 ± 6	ns^d^
GLS	−18.7 [−22.6 to −10.0]	−20.1 [−22.6 to −14.8]	−17.0 [−21.0 to −10.0]	−18.4 [−22.6 to −15]	−18.5 [−22.1 to −14.8]	−19.2 [−20.0 to −16.5]	ns^d^
**TTE diastolic function**							
LA volume/BSA (ml/m^2^)	16.1 ± 3.9	16.0 ± 3.4	17.9 ± 4.2	15.3 ± 4.0	14.4 ± 4.2	17.0 ± 2.3	ns^d^
MV E/A ratio	1.6 [1.0–3.3]	1.4 [1.0–2.1]	1.6 [1.0–3.1]	1.6 [1.0–3.3]	1.3 [1.0–3.0]	1.7 [1.5–2.3]	ns^d^
MV E dec (ms)	175 ± 37	179 ± 47	171 ± 32	183 ± 32	162 ± 33	167 ± 40	ns^d^
MV E/E' sept	6.0 [3.4–17.2]	5.3 [3.8–7.6]	6.9 [3.8–17.2]	5.3 [3.4–7.7]	7 [5.4–15.0]	6.5 [3.7–8.4]	0.006^d^
MV E/E' sept abnormal;	8/72 (11)	0/18	4/15 (27)	0/23	3/10 (30)	1/6 (17)	0.001^e^
*n/N* (%)							
**CMR**
LGE positive;	0	0	0	0	0	0	NA
*n/N* (%)							
T1 ECV	22 ± 2	20	22 ± 2	NA	21 ± 1	25 ± 1	ns^d^
**Any cardiovascular abnormality**							
*n/N* (%)	38/79 (48)	9/20 (45)	14/17 (82)	5/24 (21)	5/10 (50)	5/8 (63)	0.003 ^e^

*Data are shown as mean ± standard deviation or median [range] according to data distribution*.

* *Other oncologic diagnosis include nephroblastoma (N = 4), neuroblastoma (N = 3), and hepatoblastoma (N = 1)*.

a *NTproBNP elevated defined by values >100 ng/dL*.

b *Myocardial hypertrophy defined by left ventricular enddiastolic posterior wall z-score >2*.

c *Left ventricular dilatation defined by left ventricular enddiastolic diameter z-score >2*.

d *ANOVA (analysis of variances) or Kruskall-Wallis-test according to data distribution*.

e *Pearson chi square*.

*N, number of patients; ns, not significant; NA, not available; ng, nanogram; dL, deciliter; CPET, cardiopulmonary exercise testing; VO_2_ max (% norm), peak respiratory oxygen uptake in % predicted; NTproBNP, N-terminal pro-brain natriuretic peptide; cTNT, cardiac troponin T; TTE, transthoracic echocardiography; LV, Left ventricular; EF, Ejection Fraction; GLS, Global longitudinal strain; LA, left atrium; BSA, body surface area; MV, Mitral valve; CMR, cardiovascular magnetic resonance imaging; LGE, Late gadolinium enhancement; ECV, extracellular volume fraction*.

There was no influence of age of diagnosis, age at follow-up and follow-up time on cardiac features on Pearson's correlation in univariate analysis.

**Figure 2:**
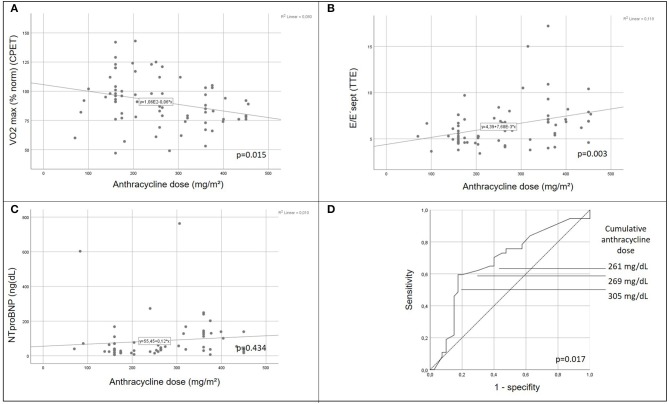
Correlation of cardiomyopathic changes with anthracycline dose. Peak respirator oxygen uptake on cardiopulmonary exercise testing **(A)** decreases and septal mitral valve E/E' ratio **(B)** increases significantly with increasing cumulative anthracycline dose (*p* = 0.015 and *p* = 0.003, respectively, pearson chi square). Trend to increased NTproBNP values with increasing cumulative anthracycline dose **(C)**. Receiver operating characteristic (ROC) curve illustrating the ability of cumulative anthracycline dose predicting any cardiovascular abnormality (decreased exercise capacity, increased NTproBNP level, or abnormal tissue Doppler septal E/E'ratio) on follow-up **(D)**. CPET, cardiopulmonary exercise testing; VO_2_ max (% norm), peak respiratory oxygen uptake in % predicted; EF, Ejection Fraction; TTE, transthoracic echocardiography.

**Figure 3:**
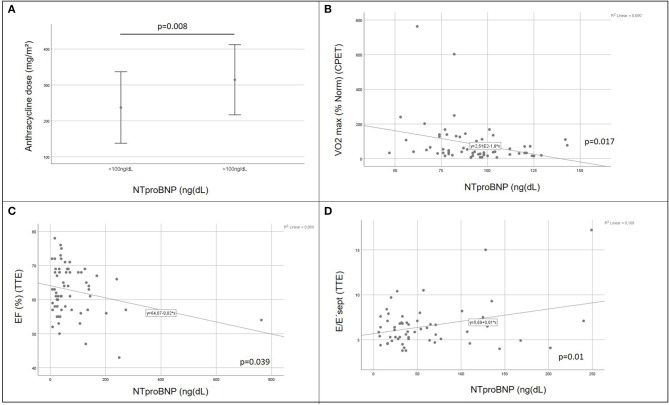
Role of the laboratory biomarker NTproBNP in cardiovascular impairment of childhood cancer survivors. Patients with elevated NTproBNP values were exposed to significantly higher doses of cumulative anthracycline compared to patients with NTproBNP values within the normal range [**(A)** independent t-Test 0.008]; increasing NTproBNP values correlated with decreasing exercise capacity [VO_2_ max (% norm), **(B)** Pearson correlation *p* = 0.017], decreasing echocardiographic systolic left ventricular function [EF, **(C)** Pearson correlation *p* = 0.039], and increasing echocardiographic diastolic dysfunction parameters [tissue Doppler septal E/E'ratio, **(D)** Pearson correlation *p* = 0.01]; CPET, cardiopulmonary exercise testing; VO_2_ max (% norm), peak respiratory oxygen uptake in % predicted; EF, Ejection Fraction; TTE, transthoracic echocardiography.

There was a positive correlation between increasing cumulative anthracycline doses and decreased exercise capacity on CPET (Figure 2A). Septal E/E' ratio on pulse wave and tissue Doppler echocardiography as a correlate of diastolic function increased significantly with increasing cumulative anthracycline doses (Figure 2B). There was a trend toward increased NTproBNP levels on Pearson correlation (Figure 2C), and patients with increased NTproBNP levels had received a significantly higher cumulative anthracycline dose compared to patients with normal NTproBNP levels Figure 3A). Increasing NTproBNP values correlated with decreasing exercise capacity, decreasing echocardiographic systolic left ventricular function, and increasing echocardiographic diastolic dysfunction parameters (Figures 3B–D). Patients with abnormal cardiovascular findings were exposed to a significantly higher amount of cumulative anthracycline compared to patients with normal evaluations (cumulative anthracycline dose 294 ± 105 mg/m^2^ vs. 231 ± 95 mg/m^2^, *p* = 0.007, independent *t*-Test).

There was no significant correlation between cumulative anthracycline dose and decreased systolic echocardiographic function as shown by TTE ejection fraction (EF) on follow-up.

There was no correlation between exposure to mediastinal irradiation or exposure to total body irradiation and abnormal cardiac findings in univariate analysis. Patients with relapse ALL who were all exposed to mediastinal radiation in the setting of TBI for bone marrow transplantation conditioning had also received higher cumulative anthracycline doses, so that mediastinal radiation exposure was eliminated as independent predictor on multivariate analysis (see below).

### Multivariate Analysis and Identification of Predictors for Risk Stratification

Increased cumulative anthracycline dose during cancer treatment was the only independent predictor for any cardiovascular abnormality (decreased exercise capacity or elevated NTproBNP or evidence of diastolic dysfunction) after elimination of influencing factors, such as oncologic diagnosis, gender, age at diagnosis, age at follow-up, follow-up time or exposure to irradiation, on multivariate logistic regression analysis (*p* = 0.036). Cut-off levels of the cumulative anthracycline dose associated with any cardiac abnormality on follow-up examination on the ROC-curve were 261, 269, and 305 mg/dL for a true positive prediction rate (sensitivity) of 62.2, 59.5, and 54.1%, and a false-positive prediction rate (1- specificity) of 30, 20, and 17.5%, respectively (Figure 2D).

## Discussion

This study reports the results of a detailed cardiovascular examination in a large group of clinically asymptomatic childhood cancer survivors on long-term follow-up. All patients were in NYHA functional class I and had a normal echocardiographic ejection fraction. Subclinical cardiovascular disease was evident by increased circulating NTproBNP levels, decreased exercise capacity, increased global longitudinal strain on echocardiographic speckle tracking, and evidence of diastolic dysfunction on echocardiographic tissue Doppler. The only independent predictor for any abnormal cardiovascular finding identified on multivariate analysis was the amount of cumulative anthracycline dose administered during cancer treatment. Major findings include the absence of overt clinical signs but the presence of subtle impairment of cardiovascular function most prevalent in patients late after successful treatment of sarcoma, as well as a correlation of these subclinical findings to the exposure of higher amounts of anthracycline during cancer treatment. When comparing patients with abnormal cardiovascular findings to those without, the cumulative anthracycline dose was close to 300 mg/m^2^ in those who showed cardiovascular impairment. The data support a threshold of >300 mg/m^2^ as critical cumulative anthracycline dose that puts patients at a higher risk for later myocardial remodeling (59–62).

No correlation was found between mediastinal irradiation exposure and abnormal cardiovascular findings in the current study, although this has previously been suggested by others (8, 9, 63). Two thirds of patients with Hodgkin disease were exposed to mediastinal irradiation, but their cumulative anthracycline dose was lower compared to the other groups and they displayed the lowest amount of any cardiovascular abnormalities. Cardiovascular abnormalities occurred more frequently in patients with relapse ALL who were all exposed to mediastinal irradiation. However, those patients had also received higher doses of cumulative anthracycline because of recurrence of disease. Since the current analysis did not reveal any correlation with abnormal cardiovascular findings and exposure to mediastinal irradiation after correction for cumulative anthracycline dose, gender, age, height and weight differences, the patients were evaluated as one group to assess correlation with anthracycline dose. According to others (63) there is a high risk of developing cardiomyopathy if >15 Gray of mediastinal irradiation is given in addition to >100 mg/m^2^ of cumulative anthracycline doses. Not detecting an aggravating effect from radiotherapy in the current study may be because the time of 11.2 ± 4.5 years from cancer diagnosis to the follow-up cardiac examination was rather short, since deteriorating effects from mediastinal irradiation are expected to occur at a later point in time (8).

The reported positive correlation of decreased exercise capacity on CPET with increasing cumulative anthracycline dose and increasing NTproBNP levels contributes to the large body of evidence about impaired exercise capacity in childhood cancer survivors (64–66) and supports the value of CPET in identifying subclinical changes (67).

The role of circulating biomarkers as predictors for subclinical cardiovascular disease in childhood cancer survivors has been suggested by others (68). Serum cardiac troponins are widely used in assessing myocardial damage in adults and children (69). No patient had elevated cardiac troponin T (cTNT) levels in the current study providing evidence for the absence of any acute ischemic or clinically overt myocardial disease. Thus, cardiac troponin T does not seem suitable to detect cardiac sequelae late after childhood cancer treatment. In contrast, NTproBNP levels were increased in patients exposed to high cumulative anthracycline doses even in the absence of clinical signs of heart failure or decreased ejection fraction on echocardiography. NTproBNP is elevated in response to ventricular wall stress (70) and increased levels have been shown as marker for diastolic dysfunction or myocardial stress (71, 72). The results of the current study together with other data (68) support the role of circulating serum NTproBNP levels as biomarkers for subclinical cardiovascular impairment in asymptomatic childhood cancer survivors.

On transthoracic echocardiography, there were no abnormalities in morphologic parameters, such as myocardial wall thickness, left ventricular enddiastolic dimensions or left atrial volumes in the patients studied, supporting the evidence of subclinical rather than clinical cardiovascular impairment. However, patients after treatment for sarcoma and most exposure to anthracyclines had lower enddiastolic left ventricular dimension and thinner posterior walls compared to the other groups. This was observed by others (73, 74) and supports the theory of myocardial wall thinning secondary to progressive cardiomyocyte loss after cardiotoxic cancer treatment (75, 76). The observations of the current study are supported by the comparison with a cohort of childhood hypertrophic cardiomyopathy patients which shows fundamental differences between the two types of cardiomyopathy.

Because myocardial and interstitial fibrosis occurs in childhood hypertrophic cardiomyopathy patients (77) and has been described in histopathological specimens obtained at autopsy of childhood cancer survivors (78) and in adult patients with anthracycline-induced decreased left ventricular function poor LV function (79, 80), CMR was performed in a subset of the studied cohort. There was no evidence of myocardial fibrosis in the studied cohort, suggesting that myocardial fibrosis is rather a sign of late disease and not evident in subclinical disease status. However, the prevalence of myocardial fibrosis and its impact on rhythm disorders or ventricular dysfunction in adult childhood cancer survivors >30 years of age cannot be assessed based on the present data because of the follow-up time reaching to young adulthood only. Based on those findings it can be concluded that CMR studies can be preserved to those patients with overt cardiac dysfunction on echocardiography or to specific questions.

As shown in other studies (81), global longitudinal strain on echocardiographic speckle tracking was impaired in about a third of childhood cancer survivors in the current study in the absence of a decreased ejection fraction, supporting the use of speckle-tracking echocardiography to detect subtle changes in cardiac dysfunction. However, speckle tracking echocardiography needs to be performed offline and is time-consuming, thus elevating costs during regular patient follow-up.

In the absence of enlarged left atria, the ratio of mitral valve septal annular E to the early velocity as measured by tissue Doppler imaging (E') (E/E') was increased in patients with subclinical cardiovascular impairment in the current study, correlating with the amount of cumulative anthracycline exposure as a sign of subclinical diastolic dysfunction. This was also shown by others (67, 82–84) and supports the hypothesis of a progressive, restrictive-like cardiomyopathy pattern previously described in serial echocardiographic studies of long-term childhood cancer survivors treated with anthracyclines (59, 74, 85–88).

In summary the presented data provide evidence of subclinical cardiovascular disease in childhood cancer survivors exposed to high doses of anthracyclines. Subtle changes may precede clinical manifestation and can be evaluated by laboratory testing (biomarkers) and focused echocardiographic imaging techniques including speckle tracking and tissue Doppler.

Of course, focus should always be on the primary prevention of cardiotoxicity (89) by limiting cumulative dosage, using liposomal anthracyclines (31–33), and providing adjunctive cardioprotective therapies (34–37) [specifically the iron-chelating agent dexrazoxane (90–92)]. On the other hand, patients at risk may benefit from early detection of subclinical heart failure in order to start preventative cardiovascular measures in time (74, 93–96).

## Conclusion

Long-term childhood cancer survivors exposed to cardiotoxic therapy show subtle impairment of cardiovascular function best detected by decreased exercise capacity, elevated NTproBNP levels, increased global longitudinal strain on speckle tracking, and abnormal mitral valve annular septal tissue doppler velocities in the absence of gross morphological changes, such as myocardial fibrosis on CMR, or echocardiographic decreased ejection fraction, enlarged left ventricular chamber sizes or left atria. Increased cumulative anthracycline dose for cancer treatment is the only independent predictor for the presence of those abnormal cardiovascular findings. Regular application of blood biomarkers and new imaging technologies can optimize risk stratification of childhood cancer survivors and can facilitate identification of subclinical cardiovascular disease and enable preventative treatment measures in time before overt clinical disease in high risk childhood cancer patients.

## Data Availability Statement

The raw data supporting the conclusions of this article will be made available by the authors, without undue reservation, to any qualified researcher.

## Ethics Statement

The studies involving human participants were reviewed and approved by the Technical University of Munich Institutional Review Board (ethical approval number 243/17S, 10/16/2017). Written informed consent from the participants' legal guardian/next of kin was not required to participate in this study in accordance with the national legislation and the institutional requirements.

## Author's Note

Data were in part presented at the Annual Meeting of the Association for European Pediatric and Congenital Cardiology in Athens, 9 May – 12 May 2018.

## Author Contributions

CW, RO, PE, IS, and JW designed the study and data collection instruments, coordinated and supervised data collection, statistical analysis and interpretation of data, drafted, reviewed and critically revised the manuscript. BR, AK, AH, JM, and CM collected cardiovascular data. BR and AH analyzed cardiopulmonary exercise testing data. AK and AH performed measurements on transthoracic echocardiography, specifically of diastolic dysfunction on tissue Doppler and speckle tracking for global longitudinal strain analysis. CM performed and analyzed cardiovascular magnetic resonance imaging and contributed to interstitial fibrosis quantification by determination of extracellular volume fraction on T1 map as well as quantification of late gadolinium enhancement and critically revised the article for important intellectual content regarding cardiovascular magnetic resonance imaging. IS collected oncologic data. CW carried out statistical analyses and drafted the manuscript. All authors listed have made substantive intellectual contributions to this study. Each author has provided final approval of the version to be published and has given agreement to be accountable for all aspects of the work in ensuring that questions related to the accuracy or integrity of any part of the work are appropriately investigated and resolved.

### Conflict of Interest

The authors declare that the research was conducted in the absence of any commercial or financial relationships that could be construed as a potential conflict of interest.

## Abbreviations

HCM: Hypertrophic cardiomyopathy
TTE: Transthoracic echocardiography
ECG: Electrocardiogram
CPET: Cardiopulmonary exercise testing
CMR: Cardiovascular magnetic resonance
LVPWd: Enddiastolic left ventricular posterior wall dimension
LVEDd: Left ventricular enddiastolic diameter
EF: Ejection Fraction
GLS: Global longitudinal strain
LA: Left atrium
MV: Mitral valve
LGE: Late gadolinium enhancement
MOLLI: Modified look-locker inversion recovery sequence
ECV: Extracellular volume fraction
NTproBNP: N-terminal pro-brain natriuretic peptide.
